# Correction: Reinforcing pomelo peel pectin beads with l-glutamate-modified graphene oxide for high-stability BOD biosensing

**DOI:** 10.1039/d6ra90044e

**Published:** 2026-05-08

**Authors:** Boi An Tran, Vy Thi Tuong Nguyen, Ngoc Xuan Nguyen, Thi-Kim-Chi Huynh, Thanh-Linh Huynh Duong, Thanh-Quang Le, Hoang-Duy Nguyen, Thi-Kim-Dung Hoang

**Affiliations:** a Institute of Advanced Technology, Vietnam Academy of Science and Technolgy No. 1B Thanh Loc 29 Street, An Phu Dong Ward Ho Chi Minh City Vietnam htkdung@iat.vast.vn; b Faculty of Applied Sciences, Ton Duc Thang University No. 19 Nguyen Huu Tho Street, Tan Hung Ward Ho Chi Minh City 70000 Vietnam tranboian@tdtu.edu.vn tranboian@gmail.com

## Abstract

Correction for ‘Reinforcing pomelo peel pectin beads with l-glutamate-modified graphene oxide for high-stability BOD biosensing’ by Boi An Tran *et al.*, *RSC Adv.*, 2026, **16**, 20498–20513, https://doi.org/10.1039/D6RA00695G

The authors regret that one of the affiliations (affiliation *b*) was incorrectly shown in the original manuscript. The corrected list of affiliations is as shown here.

The Royal Society of Chemistry apologises for these errors and any consequent inconvenience to authors and readers.

